# Nurses’ lifestyle behaviours, health priorities and barriers to living a healthy lifestyle: a qualitative descriptive study

**DOI:** 10.1186/s12912-014-0038-6

**Published:** 2014-11-28

**Authors:** Lindokuhle P Phiri, Catherine E Draper, Estelle V Lambert, Tracy L Kolbe-Alexander

**Affiliations:** UCT/MRC Research Unit for Exercise Science and Sports Science, Department of Human Biology, Faculty of Health Sciences, University of Cape Town, Cape Town, South Africa; Centre for Research on Exercise, Physical Activity and Health; School of Human Movement Studies, University of Queensland, Queensland, Australia

**Keywords:** Nurses’ health, Lifestyle behaviours, Perceptions, Shift workers

## Abstract

**Background:**

Nurses have an increased risk for non-communicable diseases (NCDs), along with a high prevalence of obesity, poor eating habits and insufficient physical activity. The aim of this study was to determine the health concerns, health priorities and barriers to living a healthy lifestyle among nurses and hospital management staff from public hospitals in the Western Cape Metropole, South Africa.

**Methods:**

Participants were purposively sampled (n = 103), and included management personnel (n = 9), night shift (n = 57) and day-shift nurses (n = 36). Twelve focus groups (FGDs) were conducted with nursing staff to obtain insight into nurses’ health concerns, lifestyle behaviours and worksite health promotion programmes (WHPPs). Seven key informant interviews (KII) were conducted with management personnel, to gain their perspective on health promotion in the worksite. Thematic analysis was used to analyse the data with the assistance of Atlas.ti Qualitative Data Analysis Software.

**Results:**

Night shift nurses frequently identified weight gain and living with NCDs such as hypertension as their main health concerns. Being overweight was perceived to have a negative impact on work performance. All nurses identified backache and exposure to tuberculosis (TB) as occupation-related health concerns, and both management and nurses frequently reported a stressful working environment. Nurses frequently mentioned lack of time to prepare healthy meals due to long working hours and being overtired from work. The hospital environment was perceived to have a negative influence on the nurses’ lifestyle behaviours, including food service that offered predominantly unhealthy foods. The most commonly delivered WHPPs included independent counselling services, an online employee wellness programme offered by the Department of Health and wellness days in which clinical measures, such as blood glucose were measured. Nurses identified a preference for WHPPs that provided access to fitness facilities or support groups.

**Conclusions:**

Public hospitals are a stressful work environment and shift work places an additional strain on nurses. The risk of NCDs and exposure to infectious disease remains a concern in this working population. Our findings highlight the need for WHPPs that support nurses in managing stress and transforming the work environment to facilitate healthy lifestyles.

## Background

The workplace is defined as an environment in which workers and managers collaborate to promote the health and wellbeing of all workers [1]. Also, the worksite is internationally recognized as an appropriate setting for health promotion and disease prevention [2] as this is where working individuals could spend up to 60% of their waking hours [3]. Employees including nurses are at increased risk of non-communicable diseases (NCDs) like diabetes, hypertension and coronary heart diseases (CHD)[4]. The main risks of NCDs are physical inactivity, unhealthy eating, smoking and alcohol abuse [5].

NCD risk factors such as physical inactivity and comorbidities like obesity have been widely reported among nurses in countries like Australia, United Kingdom, New Zealand and South Africa [4,6-9]. Similarly, nearly one-fifth of the South African healthcare workers, including doctors, dentists, nurses, radiographers, physiotherapists and occupational therapists, reported having NCDs such as hypertension and diabetes [4]. In addition, more than 70%, are overweight or obese [4]. Also, overweight participants experienced a higher prevalence of diseases and health problems than those with a normal body mass index (BMI) [4].

Additionally, Naidoo R et al., reported poor physical activity levels among nurses in KwaZulu-Natal, South Africa [10]. These findings are supported by other studies [6,9] which found that nurses do not meet the recommended levels of physical activity required for the benefit of health (30 minutes, 5 days a week). Other behavioural risk factors that have been identified among nurses include smoking and alcohol abuse [11-14].

In response to these problems, several research findings have emphasized the need for worksite wellness programmes to improve nurses’ health and lifestyle behaviours, including physical activity [15,16]. A three-month physical activity intervention in nurses showed significant differences in Body Mass Index (BMI) from pre to post intervention [15]. Similarly, a 10 weeks physical activity intervention study in hospital-based registered nurses showed significant effects on fat mass, fat index and fat percentage (*p* <0 .03) [16]. The intervention participants’ fat mass decreased from 28.4 to 27.8 and the fat mass percentage decreased from 39.1% to 38.4%. [16]. These findings are supported by a workplace physical activity interventions meta-analysis by Conn VS et al. [17] who concludes that some workplace physical activity interventions can improve health and important worksite outcomes.

Because patient care cannot be confined to usual working hours (09h00 – 17h00), approximately a quarter of all nurses work non-traditional hours or shifts [18,19]. Shift work can have a negative impact on the employee and could lead to increased drug use, job-related stress, poor job performance, insomnia, and disrupted social and family life [19-23]. The high prevalence of health-related conditions and risk factors such as obesity, overweight, physical inactivity, and poor eating habits has been reported amongst shift and rotational night shift workers [24-28].

Therefore, the main aim of this research study was to explore the health priorities, current lifestyle behaviours and barriers to living a healthy lifestyle among nurses working in public hospitals in the Western Cape Metropole, South Africa. These findings will then be used to provide recommendations for a worksite wellness intervention programme for nurses aimed at reducing NCDs risk factors such as obesity, physical inactivity and poor eating habits.

## Methods

### Design and setting

This study used qualitative research methods [29] for data collection which included focus group discussions (FGDs) and key informant interviews (KIIs). The aim of the focus groups was to explore and obtain in-depth information about the nurses’ lifestyle behaviours, health concerns and priorities and barriers to living a healthy lifestyle. These included lifestyle behaviours such as habitual levels of physical activity, smoking and dietary habits. In addition, the preferred types of WHPPs and factors that might influence their participation in the WHPPs were also investigated. Seven key informant interviews were conducted with management personnel in order to gain their perspective on health promotion in the worksite.

### Setting

**Figure 1:**
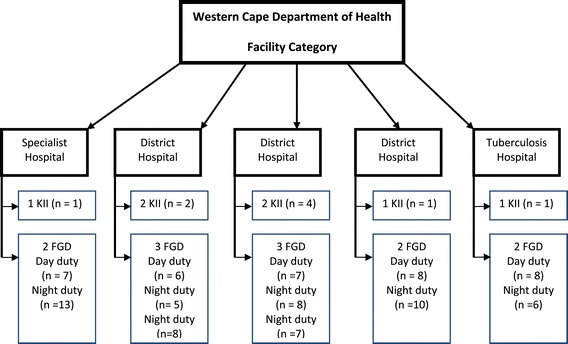
**Facility category classification by the Western Cape Province Department of Health (KIIs and FGDs).**

As shown in Figure 1, all hospitals in the Western Cape Metropole were eligible to participate in the study. There are a total of 22 public hospitals in this region, including three tertiary hospitals, two specialist hospitals, nine district hospitals, four psychiatric hospitals, one regional hospital and one tuberculosis hospital. Half of the hospitals were purposively sampled to participate in the research study. Of the eleven hospitals invited to participate in the research study, five agreed, and these included three district hospitals, one specialist hospital, and one tuberculosis hospital.

### Participants

The Human Resources Manager and/or Nursing Manager at each hospital was invited and informed of the study via an e-mail, followed by a telephone call. This was followed by a meeting between the nursing manager and the researchers to make arrangements for the focus groups and interviews. Purposive sampling was used to select the nurses for the focus groups, which included nurses from various occupational ranks (professional nurse, staff nurse and nurse management) and hospital wards. According to the South African Nursing Council [30], a professional nurse needs to hold a University degree whereas a staff nurse holds a National Diploma in nursing. A professional nurse is competent to practice comprehensive nursing, assumes responsibility and accountability for independent decision making [30].On the other hand a staff nurses is a generalist nurse who’s practice is focused on quality service delivery within a broad spectrum of health services and in a variety of settings [30].

A total of ninety-three (n = 93) nurses, representing both night shift (n = 57) and day shift (n = 36), participated in the study. Five focus groups were conducted with the day staff, while seven focus groups were conducted with the night staff from the five participating hospitals. The group discussions comprised of a minimum of five nurses and a maximum of thirteen nurses per group. None of the hospital managers were invited to attend any of the focus group discussions in order to ensure that the nurses were free to express their opinions. This was to try to eliminate the potential negative impact of any power dynamics between the nurses and management, which could occur due to the hierarchical structures in the South African health care setting [31]. A poor interpersonal relationship between supervisors/management and nurses has been reported in a South African setting [31].

The key informant interviewees were also purposively selected, and included individuals working in a managerial role. Key informants are defined as those individuals who are knowledgeable in a specific field and/or hold special skills [32,33]. These types of informants voluntarily share their knowledge and skills, observations and insights to which the researcher would otherwise not have access [32,33]. Interviews were conducted on a one-on-one basis with seven with seven hospital managers and the seventh key informant interview was conducted with three managers. This was due to time constrains reported by these managers. The researchers acknowledge that there might be some disadvantages in having more than one manager present at the last interview, like managers not feeling free to voice their opinions.

### Data collection

The focus groups and interviews were conducted by a trained facilitator using guided questions. These included questions such as: ‘What are your main personal health concerns?’ , ‘What are the main health concerns in your workplace?’ and ‘How does your work affect your lifestyle behaviours and health?’ (Table 1). All the FGDs and KIIs were conducted within the health facility during working hours and were audio recorded with the participants’ consent. They were approximately 60 minutes in duration.

**Table 1:** **Focus groups discussion and key informant interviews guide questions**

**Number**	**Question**
1	What are your main health concerns?
2	How does your work affect your lifestyle behaviours and health?
3	What do you think the main health concerns are in your workplace?
4	How do you think your health could affect work performance?
5	Has your workplace ever implemented a wellness or worksite health promotion programme?
6	What type of programme was delivered?
7	Which factors would influence your participation in a worksite health promotion programme?
8	Which types of programmes would you like to have implemented at your workplace?
9	What is the ‘health’ and lifestyle behaviour culture among employees?
10	Which factors influence your health behaviour both at work and away from work?

### Qualitative data analyses

All focus groups and interviews were transcribed by an independent transcriber. Transcripts were analysed using thematic analysis [29], with the assistance of Atlas.ti Qualitative Data Analysis Software (Scientific Software Development GmbH, Berlin, Germany). The researcher conducting the analyses (LP) familiarised herself with the transcribed data by reading through the transcripts numerous times with the aim of identifying main themes and sub-themes, which are based on meaningful categories of data and “repeated patterns of meaning” [29].

The guide questions also informed the identification of themes in the analysis process, as these questions referred to the main issues that needed to be covered in the focus groups and interviews. Other authors (CED and TKA) were involved in the development of the guide questions. The themes and sub-themes then formed the basis of the coding framework, which was refined through consultation with the other authors (CED and TKA). This coding framework was then systematically applied to the transcripts, and portions of the text were assigned to various sub-themes. Quotes for each sub-theme were then collated and summarised, and pertinent quotes have been included in this manuscript in order to best illustrate these sub-themes. The sub-themes and themes were used to develop a conceptual framework, which also aims to summarise the data.

### Ethical consideration

The University of Cape Town Research Ethics Committee of the Faculty of Health Science (REC REF: 212/2012) approved this study. In addition, the Western Cape Department of Health provided approval for the research study to be conducted in their hospitals (Ref No: 2012; RP121). All participants gave written consent prior to participating in the focus groups and interviews.

## Results

**Figure 2:**
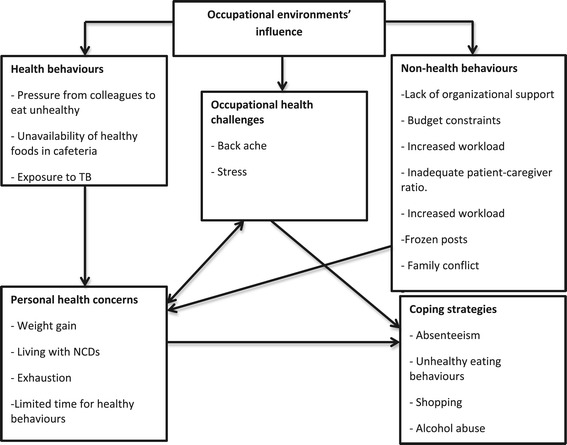
**Conceptual model summary of the results and how the different factors influence the nurses’ lifestyle behaviours.**

We have prepared a conceptual model based on the results of this study (Figure 2). The model represents a summary of the themes and sub-themes from both the focus group discussions and key informant interviews. Furthermore, this model illustrates the relationship between the various main themes and sub-themes and how these themes influence the nurses’ lifestyle behaviours and health priorities.

### Perceptions of health

Most of the management personnel (n = 6) perceived a healthy lifestyle as a good state of health. A good state of health was described as the absence of disease, being physically, spiritually and emotionally fit to cope with the challenges of the daily life. Night shift nurses reported that being healthy included eating a balanced diet, such as eating breakfast in the morning.

*“Proper meals with the right nutrition’s like you start with your breakfast which is an important meal of the day”. (FGD)*

### Personal health concerns

Weight gain and living with NCDs such as hypertension and diabetes were frequently reported by night shift nurses as their main personal health concerns. Nurses from both day and night shifts also acknowledged that overweight and obesity were a common health threat. Furthermore, being overweight was perceived to have a negative impact on work performance. Nurses mentioned that some of their overweight colleagues found it difficult to cope with their job demands.

*“Many of the nurses are overweight, too slow in the wards because they are carrying that weight ‘..’. I was looking at some of us and thought oh my, if there’s going to be an emergency will you be able to run”.* (KII)

*“Well, if they have to pick up patients and they can hardly move themselves around ‘…’ ,it’s not that easy. So people who are overweight, I think, cannot do physical work like leaner people”.* (KII)

### Occupational health challenges

Being exposed to tuberculosis (TB), including multi-drug resistance TB (MDR-TB) and extensively drug-resistant TB (XDR-TB) and the fear of contracting this infectious disease emerged as the main occupational health-related challenges for both day and night shift nurses. However, this was a greater concern for night shift nurses compared to day shift nurses.

*“When they come in casualty (accident and emergency unit) you don’t know, and you treat the patient and they are coughing and everything, but now if they come from the clinic and there is a slight hint, we give them a mask. Otherwise you don’t know what it could be and next thing you hear, oh!, someone has got TB and you like you very exposed to that”.* (FGD)

Musculoskeletal pain such as backache was identified as another occupational health concern by both day and night duty nurses. Lifting heavy patients and standing for long hours were reported as the main cause of backache among nurses.

*“In ICU ‘…’, backaches because we lift a lot of heavy patients and most of our patients are sometimes unconscious or they are heavy sedated. The physio comes she asks if we can help to lift the patient ‘…’, the x-ray lady also comes and asks if we can help lift the patient. The doctor comes and wants to listen to the chest, and also ask if you can help to lift the patient. We do pressure care like four hourly or six hourly, we need to lift the patient and turn the patient and then we need to turn the patient to the other side twice”.* (FGD)

### Influence of the occupational environment on health and lifestyle behaviours

Nurses frequently mentioned lack of time to prepare healthy meals due to long working hours and being overtired from work as major challenges to leading a healthy lifestyle. Buying fast foods was regarded as the most convenient option, and in most cases fast foods were unhealthy. Some of the day and night shift nurses reported that occupational stress was one of the reasons they ate throughout the day. Interestingly, the fear of contracting TB was another reason mentioned for eating throughout the day as the nurses believed that it is easier to contract the TB on an empty stomach as your immune system is not strong enough to handle the infections.

*“Eating all the time, I especially eat when I’m stressed, I eat when I’m happy then ‘…’, like I’m working in C ward, especially during the day then you don’t even have time to have, for tea, or lunch time, then you just grab anything you know. Eating in a rush, that stresses me*”. (FGD)

Both management and day shift nurses agreed that the hospital cafeterias sold predominantly unhealthy foods such as deep-fried chicken, hot chips (french fries) and pies (pastries). Some of the reasons for choosing less healthy foods were related to cost, as healthier foods like fruits and salads were more expensive in the cafeteria. A small number of the management and day and night shift nurses were of the opinion that the lifestyle culture in the worksite was neither healthy nor unhealthy. This is because some nurses preferred eating fruits, while others preferred eating deep-fried hot chips (french fries). There was little input about the cafeteria services from the night shift nurses as it was reported that the cafeteria was closed at night.

*“You know there is not one healthy thing at that tuck shop other than the little bowl of fruit, and that is why the girls will buy the chips and the coke and whatever. That tuck shop should be scrapped or healthy”.* (FGD)

Some of these nurses felt that their colleagues negatively influenced their health behaviours by making them feel guilty for choosing not to eat cake on certain days. Others felt that their colleagues were a good influence as they encouraged them to have a healthy diet and also gave advice on the healthy food choices.

*“Around our department there is always cake and stuff like that and if you say no, they say what is wrong with you, so you just like obliged to say okay I will also have another piece, so they are encouraging me not to be healthy”.* (FGD)

Perceived lack of support from the Department of Health (government), burnout, needle stick injuries and insufficient and poor quality resources such as aprons and gloves, were identified as occupational environmental safety challenges by day and night shift nurses. Torn gloves can expose nurses to diseases that can be contracted through bodily fluids, like Hepatitis B. It was also reported that needle stick injuries were more prevalent among night shift nurses and often occurred in the early hours of the morning when the nurses were exhausted. In contrast, burnout was frequently reported by the day shift nurses and not night shift nurses.

*“And funny enough my observation has been that most of the needle injuries that have been in theatre are when people are exhausted at that time of the morning, two, three am”.* (FGD)

Besides personal and occupation-related health and safety challenges, there was an emergence of other non-health occupational related challenges experienced by the nurses. These included lack of recognition for hard work from management which was frequently reported by night shift nurses. Another non-health occupational related challenge frequently reported by the day shift nurses and management was the hiring of agency nurses. Agency nurses were described as nurses that are temporarily hired by the Department of Health to assist with the shortage of nurses. However, this temporary solution appears to add to the nurses’ occupational-related stress, as they reported that they had limited time to educate the agency nurses about the patients’ required treatment and hospital procedures.

*“I’m not unhappy, but I can tell you if I’ve got eight patients to look after and there is an agency nurse that knows nothing. She knows her observations but she doesn’t know anything and I have to see to that also, at the end of the day you are not feeling happy about it”.* (FGD)

Budget constraints within the public health system reported by nursing managers and nurses seemed to be the major contributing factor to the increased shortages of nurses. This led to increased workloads due to inadequate patient to nurse ratios. The shortage of nursing staff frequently reported by day shift nurses was perceived to be associated with the stressful working environment.

*“Because of money, they don’t have enough money to hire people, but they have money to give to agencies that’s a problem, or maybe if you are permanent you must have benefits, hence you use extra money. But, there are vacant posts”.* (FGD)

Two of the seven management personnel interviewed shared their concerns relating to alcohol abuse by nurses. Factors contributing to this abuse included financial stress and the “slow vehicle of the state”, which referred to slow government systems that hinder the filling of the vacant nursing posts, hence perpetuating the shortage of nurses.

*“They said, this is the ratio that is being used in South Africa as a developing country and we need to function like that ‘…’, and you know that I came in to work in somebody’s place, so what has been set up here over the last 4 years, I have to continue until the new hospital. They are now planning for something better, but the vehicles of the state are very slow, you know the machinery is very, very slow and so, with work load and not enough staff you will find, personal problems of staff like alcoholism”.* (KII)

Lastly, nurses reported that their work demands negatively affected their family responsibilities. This was more frequently reported by night shift nurses who mentioned that they had limited time to spend with their families due to their long working hours (12 hours) and high work demands. In addition, night shift nurses also reported feeling moody and irritable most of the time, and this sometimes created family conflict.

*“When you get home you are frustrated because you feel sick and now you must go back to that place tonight, so you take it out on the kids, the husband and everybody else”.* (FGD)

### Coping strategies

Another concern raised by the day shift nurses was absenteeism in the workplace, which resulted in having insufficient nurses on duty. The nurses and management identified absenteeism as a coping strategy, because the nurses felt that being absent from work gave them an opportunity to rest. A stressful working environment and being unhappy at work were reasons mentioned by nurses for staying away from work. Although the nurses are allocated days off to rest, they reported that sometimes they had to work overtime to earn an extra income. The nurses regarded their salaries to be inadequate for their daily living needs and necessities.

*“Because they are working so much overtime they become tired. They are working overtime to compensate for whatever is missing from their salaries, as a result they are over tired and they stay away from work, those kinds of things. So it’s a whole lot of things”.* (KII)

Absenteeism was the most commonly identified coping strategy reported by both the nurses and management. Other coping strategies identified by the nurses included eating unhealthy foods and drinking high calorie beverages. The nurses believed that these beverages helped to reduce fatigue and enabled them to cope with their work demands. Also, a few management personnel mentioned alcohol abuse and smoking as possible coping strategies. Another coping strategy mentioned by the nurses was unnecessary shopping. A consequence of purchasing unnecessary goods was having to work overtime, as they needed to earn extra money to clear debt.

*“I only drink coffee or coke at night. I will eat like you say one meal and then you don’t eat for the rest of the night and then maybe and you drink coke, and then 5 o’clock the morning you like at that point in the morning you feel like aah ‘…’, I can drink coffee now and you have that cup of coffee at five because that is the time you feel like a zombie, especially if you are working in theatre*”*.* (FGD)

### Existing worksite health promotion programmes

An online employee wellness programme (EWP) offered by the Department of Health was the most commonly identified existing worksite health promotion programme by the nurses. This is a programme designed to assist nurses experiencing personal and work-related stress as well as financial challenges.

Wellness days were the most frequently delivered worksite health promotion programmes in the worksite. The wellness days were offered by the Government Employee Medical Scheme (GEMS), a medical insurance provided by the South African government for public servants. These were delivered once or twice a year and programmes offered during these days included voluntary counselling and testing (VCT) for human immune deficiency virus (HIV), and cholesterol, blood pressure and blood glucose tested/measured. Day shift nurses and management commented on the wellness days more often as these events usually took place during the day and were rarely offered at night.

*“And even early in the year, so twice a year we try and have wellness days with staff and then we look at blood glucose, we look at high blood pressure, we also have voluntary HIV testing ‘…’, just general and we will get a dietician to come in and speak about a healthy lifestyle, eating and things like that”.* (KII)

Furthermore, day shift nurses felt that the current health and wellness programmes focused mainly on the identification of health risk factors such as obesity. The nurses reported that the wellness clinical measures did not positively impact their lives because there was a lack of programmes implementation to address the poor health behaviours. The nurses believed that a worksite health promotion programme should include individualised feedback in order to achieve long term positive impact on the individual’s health risk behaviours or/conditions. Both day shift nurses and nursing management perceived the delivered worksite health promotion programmes as being worthwhile.

*“Yes, there is something like that, but I mean that is only once off and most of the staff just go for the freebies like .But, once those people are gone, then it’s back to square one as there is not continuity with it”.* (FGD)

Negative factors which could influence the participation in the desired worksite health promotion programmes were identified. These included lack of facilities such as showers, especially after participating in physical activities, lack of interest by the nurses, staff shortages and fatigue. Limited time was frequently reported as the main reason for not participating in the existing worksite health promotion programmes. Furthermore, due to the need to have sufficient nurses in the wards to care for the patients at all times, it would be impossible for all nurses to participate in the worksite programmes simultaneously. The nurses felt that the shortage of nurses in the wards poses a risk of subsequent legal action if something were to go wrong with a patient while they were absent from the ward.

*“You can’t just take your nurses out of the work place. Your operational requirements would require that within the wards, so to take them out of their wards during working hours is usually not a good idea because some people we have a few nurses as it is. So it would be difficult to implement that, it would have to be done in their time”.* (KII)

### Desired worksite health promotion programmes

Day shift nurses suggested support groups where they could discuss their occupational and personal challenges. These nurses also suggested physical activities such as aerobic classes and a gym in the health care facility. The gym to which they have access at an affordable price is difficult to access due to transport challenges as it is situated at the head office in the central business district (CDB).

*“It will be very nice if they could organise maybe like a small gym, where you can walk in the hospital for free”.* (KII)

There was shared interest between the day and night duty shift for self-care activities, including massage sessions. Nursing management requested other worksite health promotion programmes such as team building, information about healthy meals and addressing cultural diversity.

*“But, even if it is like a little sports room and you have five little chairs with five little those foot spa’s where everyone can just go sit there and put their feet in the spa”.* (FGD)

## Discussion

A common and important theme arising from all the nurses’ focus group discussions was concerns about being overweight and living with NCDs such as hypertension and Type 2 diabetes. Another common and important theme arising from all the nurses’ focus group discussions was musculoskeletal injuries such as backache and exposure to tuberculosis (TB), these were their main occupational health-related concerns. Both management personnel and nurses frequently reported experiencing a stressful working environment. The lack of institutional support was one of the key themes that emerged and was identified by the nurses in this study as a major barrier on their ability to prioritise their health and make healthy choices. This lack of institutional support impacted on the nurses’ ability to cope with the stresses of their occupation and resorting to coping mechanisms such as absenteeism.

The fact that the nurses were most concerned with the problems of overweight, obesity and living with NCDs such as diabetes and hypertension indicate the enormous challenges faced by this vulnerable population. These findings are supported by a study in South African HCWs which reported that one out of three HCWs were diagnosed with NCDs such as hypertension and diabetes [4]. Nurses are expected to be role models to their patients and in their communities and to lead by example [4,9,34]. However, they are challenged in this role due to poor personal health and associated risk factors, for example, being overweight [4,6-9,34] and not meeting public health physical activity recommendations [6,9].

The unavailability of unhealthy foods and high cost of healthy foods in the hospitals’ cafeterias were associated with unhealthy eating habits by the nurses in this study and may be a possible factor contributing to the problem of overweight and obesity. It can be argued that modifying external factors such as increasing the availability and lowering the cost of healthy foods in the cafeteria might promote the consumption of fruits and vegetables among the nurses [35]. This has been shown to be effective in a military setting [36].

The fear of being infected with TB was the key occupational health-related issue affecting nurses in this study. This was not surprising considering, the high prevalence of TB infection amongst HCWs in the Eastern and Western Cape provinces of South Africa [37]. Fear of being exposed to and contracting TB, MDR-TB, and XDR-TB are amongst the most commonly reported occupational stressors and health concerns by nurses in South Africa [38,39]. Furthermore, South African HCWs are reported to have five to six fold increased rate of hospital admission with MDR-TB admission when compared to non-medical staff [40]. The vulnerability to TB exposure by HCWs has been found in a number of other studies [37,40,41].

Other occupational-related concerns raised by the nurses in this study including musculoskeletal pain backache, budget constraints, burnout and increased work load due to staff shortages has been reported in other research studies [19,38,42-45]. A study investigating the association between nurses’ occupational-related injuries and illnesses long work hours among nurses in the Philippines reported that over 78 percent of the nurse’s experienced back pain [46].

Increased workloads reported by the day shift nurses in this study have also been reported by nurses in other research studies [44,47]. Working day shift was reported to be more physically demanding by female nurses in Australia as it entailed bathing and lifting of patients [47]. These nurses also reported that there was more administration work during the day shift in comparison to night shift [47].

As our research study has shown, nurses working night shift found their shift had negative influence on some aspects of their lives such as limited family time and the ability to resolve family conflicts. Nurses in this study reported having insufficient time to manage their personal and home responsibilities. Some nurses mentioned that their spouses disliked their occupation based on their opinion that the nurses brought work stress and frustrations back home. Similar findings were reported by night shift workers in Iran [48].

Shortages in nursing staff, unreliable and lack of equipment and also inadequate remuneration were some of the occupational-related challenges mentioned by the nurses in this study. These challenges are echoed by other South African nurses in the Limpopo [31] and KwaZulu-Natal provinces [38]. Furthermore, George et al. [49] also reported that low salaries and human resource shortages are some of the factors leading to the immigration of nurses from sub Saharan-Africa, thus adding to the existing shortage of nurses.

Nurses in this study perceived their salaries to be inadequate, thus often worked overtime on their days off so as to add to their existing salary. Previous research has reported that nurses’ working overtime is a trend that is commonly seen in countries where nurses earn low salaries [50]. Working overtime could possibly be one of the contributing factors to the reported absenteeism, as the need for more time to rest was one of the reasons mentioned by the nurses for staying away from work. Absenteeism was raised as a problem by the nurses in this study. The shortage of nurses leads to an increased workload, and absenteeism could be interpreted as a coping strategy among nurses, helping to maintain manageable levels of physical and psychological states [51].

Findings from this study emphasise the need for a worksite health promotion programme aimed at reducing NCD health-related conditions or risk factors such as obesity, physical inactivity and poor dietary eating habits and also reducing the fear of TB infection among the nurses. Over and above these challenges, it appeared that there was a general lack of nurses’ involvement in the planning of the existing WHPPs which seemed to be one of the contributing factors to the poor levels of participation. A participatory approach in which employees are involved in both the planning of the WHPPs and active engagement in the decision-making process is deemed necessary for the success of any health promotion program [52]. Even though the existing worksite health promotion programmes addressed the nurses’ health concerns, they did not seem to address their barriers to living a healthy lifestyle. Suggested worksite health promotion programmes by the nurses in this study included fitness facilities and massage sessions. In contrast, the delivered WHPPs comprised mainly of clinical measures and online employee assistance programmes.

There was clearly mismatch between the type of WHPPs that the employees preferred and those offered, highlighting the importance of consultation and inclusion of employees in the decision-making process. Previously published research suggests that lack of consultation and exclusion of employees in the planning and decision-making process could possibly result in lack of interest from the nurses to participate in the delivered programmes [52]. However, the active involvement of the nurses in the planning and implementation of these programmes can be viewed as a critical starting point as it may result in positive outcomes and sustainability of the programmes [52].

### Strengths of the study

The strengths of this study include the naturalistic environment within which the study was conducted. The qualitative research method provided an opportunity for capturing a range of opinions among the nurses and management personnel during the focus groups and key informants. The interviews enabled the researcher’s access to the nursing managements’ knowledge, unique perspectives, observations and insights to the nurses’ lifestyle behaviours and worksite health promotion programmes. Another strength includes the representation of the various wards and shifts in the different types of hospitals in the Cape Town Metropole. This included day and night shift workers from various wards in the different hospitals. We can argue that gathering information from the different wards, shifts and hospitals assists in the greater understanding of the extent of the challenges experienced by the nurses in the Western Cape Metropole.

To the best of our knowledge, this one of the first descriptive qualitative research studies investigating nurses’ lifestyle behaviours, health priorities and barriers to living a healthy lifestyle in South Africa. Other studies have only identified the health risk factors and made recommendations for an intervention programme [4,10].

### Limitations of the study

It was often a challenge for the night shift nurses to attend the FGDs as they reported that they were fewer nurses working at night. The research study was only conducted in the Western Cape public hospitals and not private hospitals. Also, Primary Health Care facilities were not included in the data collection, largely due to most of them having only day-time hours. Conducting the group interviews might have resulted in disadvantages such as managers not being free to voice out their opinions in the presence of fellow colleagues.

## Conclusion

The prevalence of NCDs and health-related conditions and risk factors such as obesity, physical inactivity, poor dietary habits, and substance abuse has been reported among the nursing workforce. Occupational challenges including TB, musculoskeletal injuries, budget constraints, burnout and increased work load due to staff shortages have been reported among this population. The findings presented in this study call for the urgent action of implementing WHPPs such as physical activity interventions to help reduce the prevalence of NCDs, obesity and physical inactivity among nurses.

Maintaining the health of the nurses has been recognised as being important also for maintaining the health of the patients [53]. Therefore, employers in a healthcare setting have an obligation to understand and address, where possible, the main causes of physical and psychological ill-health occurring within their organisations [54]. There is good evidence demonstrating the efficacy of WHPPs to reducing NCDs health risks and potentially decreasing the cost of health care expenditure [46,55,56]. Furthermore, WHPPs have been reported to result in increased in work productivity and increase employee loyalty [57,58].

## Abbreviations

BMI: Body mass index
CBD: Central business district
CVD: Cardiovascular diseases
EWP: Employee wellness programme
FGD: Focus group discussions
IHD: Ischaemic heart disease
HCW: Health care workers
HIV: Human immune deficiency virus
HIV: Human immune deficiency virus
KII: Key informant interviews
KZN: KwaZulu-Natal
MDR-TB: Multiple-drug-resistant TB
NCD: Non-communicable diseases
SA: South Africa
TB: Tuberculosis
UK: United Kingdom
USA: United States of America
VCT: Voluntary counselling and testing
WHO: World health orginization
WHPP: Worksite health promotion programmes
XDR-TB: Extremely drug-resistant TB

## References

[1] Burton J, World Health Organization: **WHO Healthy workplace framework and model: Background and supporting literature and practices.** 2010**:**2-123.

[2] Quintiliani L, Sattelmair J, Sorensen G (2007). The Workplace As A Setting For Interventions To Improve Diet And Promote Physical Activity.

[3] Batt ME (2009). Physical activity interventions in the workplace: the rationale and future direction for workplace wellness. Br J Sports Med.

[4] Skaal L, Pengpid S (2011). Obesity and health problems among South African healthcare workers: do healthcare workers take care of themselves?. S Afr Fam Pract.

[5] World Health Organization: Global status report on noncommunicable diseases 2010. http://www.who.int/nmh/publications/ncd_report2010/en/.

[6] Blake H, Harrison C (2013). Health behaviours and attitudes towards being role models. Br J Nurs.

[7] Bogossian FE, Hepworth J, Leong GM, Flaws DF, Gibbons KS, Benefer CA, Turner CT (2012). A cross-sectional analysis of patterns of obesity in a cohort of working nurses and midwives in Australia, New Zealand, and the United Kingdom. Int J Nurs Stud.

[8] Miller SK, Alpert PT, Cross CL (2008). Overweight and obesity in nurses, advanced practice nurses, and nurse educators. J Am Acad Nurse Pract.

[9] Blake H, Malik S, Mo PKH, Pisano C (2011). ‘Do as I say, but not as I do’: are next generation nurses role models for health?. Perspect Public Health.

[10] Naidoo R, Coopoo Y (2007). The health and fitness profiles of nurses in KwaZulu-Natal. Curationis.

[11] Aldiabat KM, Clinton M (2013). Understanding Jordanian psychiatric nurses’ smoking behaviors: a grounded theory study. Nurs Res Pract.

[12] Berkelmans A, Burton D, Page K, Worrall Carter L (2011). Registered nurses’ smoking behaviours and their attitudes to personal cessation. J Adv Nurs.

[13] Servodidio CA (2011). Alcohol abuse in the workplace and patient safety. Clin J Oncol Nurs.

[14] Smith DR (2007). Longitudinal trends of alcohol and tobacco consumption among Australian physicians and nurses, 1989-2005. J Subst Use.

[15] Yuan SC, Chou MC, Hwu LJ, Chang YO, Hsu WH, Kuo HW (2009). An intervention program to promote health-related physical fitness in nurses. J Clin Nurs.

[16] Tucker SJ, Lanningham-Foster LM, Murphy JN, Thompson WG, Weymiller AJ, Lohse C, Levine JA (2011). Effects of a worksite physical activity intervention for hospital nurses who are working mothers. AAOHN J.

[17] Conn VS, Hafdahl AR, Cooper PS, Brown LM, Lusk SL (2009). Meta-analysis of workplace physical activity interventions. Am J Prev Med.

[18] Beers TM: **Flexible schedules and shift work: replacing the 9-to-5 workday.** *Monthly Lab Rev* 2000, **123:**33.

[19] Swartz LB: **Experiencing night shift nursing: a daylight view**. University of the Western Cape, Faculty of Community and Health Sciences; 2006.

[20] Gordon NP, Cleary PD, Parker CE, Czeisler CA (1986). The prevalence and health impact of shiftwork. Am J Public Health.

[21] Shields M (2002). Shift work and health. Health Rep.

[22] Shen J, Botly LCP, Chung SA, Gibbs AL, Sabanadzovic S, Shapiro CM (2006). Fatigue and shift work. J Sleep Res.

[23] Abdalkader R, Hayajneh F (2008). Effect of Night Shift on Nurses Working in Intensive Care Units at Jordan University Hospital. Eur J Sci Res.

[24] De Bacquer D, Van Risseghem M, Clays E, Kittel F, De Backer G, Braeckman L (2009). Rotating shift work and the metabolic syndrome: a prospective study. Int J Epidemiol.

[25] LdCÃo A, Jornada MN, Ramalho L, Hidalgo MPL (2010). Correlation of shift work and waist circumference, body mass index, chronotype and depressive symptomsArq Bras Endocrinol Metabol. Arq Bras Endocrinol Metabol.

[26] Morikawa Y, Miura K, Sasaki S, Yoshita K, Yoneyama S, Sakurai M, Ishizaki M, Kido T, Naruse Y, Suwazono Y (2008). Evaluation of the effects of shift work on nutrient intake: a cross-sectional study. J Occup Health.

[27] Zhao I, Turner C: **The impact of shift work on people’s daily health habits and adverse health outcomes.** *Australian Journal of Advanced Nursing, The* 2008, **25:**8.

[28] Zhao I, Bogossian F, Song S, Turner C (2011). The association between shift work and unhealthy weight: a cross-sectional analysis from the Nurses and Midwives’ e-cohort Study. J Occup Environ Med.

[29] Braun V, Clarke V (2006). Using thematic analysis in psychology. Qual Res Psychol.

[30] South African Nursing Council. http://www.sanc.co.za/neis.htm.

[31] Kekana HPP, Du Rand EA, Van Wyk NC (2007). Job satisfaction of registered nurses in a community hospital in the Limpopo Province in South Africa. Curationis.

[32] Patton MQ (2002). Qualitative Research.

[33] Miller WL, Crabtree BF (1992). Primary Care Research: A Multimethod Typology And Qualitative Road Map.

[34] Zapka JM, Lemon SC, Magner RP, Hale J (2009). Lifestyle behaviours and weight among hospital-based nurses. J Nurs Manag.

[35] Backman D, Gonzaga G, Sugerman S, Francis D, Cook S (2011). Effect of fresh fruit availability at worksites on the fruit and vegetable consumption of low-wage employees. J Nutr Educ Behav.

[36] Bingham CML, Lahti-Koski M, Puukka P, Kinnunen M, Jallinoja P, Absetz P: **Effects of a healthy food supply intervention in a military setting: positive changes in cereal, fat and sugar containing foods.** *Int J Behav Nutr Phys Act* 2010, **9:**91. 10.1186/1479-5868-9-91 PMC3511183 22849620

[37] Jarand J, Shean K, O’ Donnell M, Loveday M, Kvasnovsky C, Van der Walt M, Adams S, Willcox P, O’ Grady J, Zumla A (2010). Extensively drug resistant tuberculosis (XDR-TB) among health care workers in South Africa. Tropical Med Int Health.

[38] King LA, McInerney PA (2006). Hospital workplace experiences of registered nurses that have contributed to their resignation in the Durban metropolitan area. Curationis.

[39] Malangu N, Legothoane A: **Analysis of occupational infections among health care workers in Limpopo Province of South Africa.** *Glo J Health Sci* 2012, **5:**p44. 10.5539/gjhs.v5n1p44 PMC4777004 23283035

[40] O’Donnell MR, Jarand J, Loveday M, Padayatchi N, Zelnick J, Werner L, Naidoo K, Master I, Osburn G, Kvasnovsky C (2010). High incidence of hospital admissions with multidrug-resistant and extensively drug-resistant tuberculosis among South African health care workers. Ann Intern Med.

[41] Joshi R, Reingold AL, Menzies D, Pai M: **Tuberculosis among health-care workers in low-and middle-income countries: a systematic review.** *PLoS Med* 2006, **3:**e494. 10.1371/journal.pmed.0030494 PMC1716189 17194191

[42] Huntington A, Gilmour J, Tuckett A, Neville S, Wilson D, Turner C (2011). Is anybody listening? A qualitative study of nurses’ reflections on practice. J Clin Nurs.

[43] Nyathi M (2008). Working conditions that contribute to absenteeism among nurses in a provincial hospital in the Limpopo Province. Curationis.

[44] Vagharseyyedin SA, Vanaki Z, Mohammadi E (2011). Quality of work life: Experiences of Iranian nurses. Nurs Health Sci.

[45] Rothmann S, Van Der Colff JJ, Rothmann JC (2006). Occupational stress of nurses in South Africa. Curationis.

[46] De Castro AB, Fujishiro K, Rue T, Tagalog EA, Samacoâ-Paquiz LPG, Gee GC (2010). Associations between work schedule characteristics and occupational injury and illness. Int Nurs Rev.

[47] West S, Mapedzahama V, Ahern M, Rudge T (2012). Rethinking shiftwork: mid-life nurses making it work!. Nurs Inq.

[48] Nasrabadi AN, Seif H, Latifi M, Rasoolzadeh N, Emami A (2009). Night shift work experiences among Iranian nurses: a qualitative study. Int Nurs Rev.

[49] George G, Rhodes B: **Is there really a pot of gold at the end of the rainbow? Has the Occupational Specific Dispensation, as a mechanism to attract and retain health workers in South Africa, leveled the playing field?** *BMC Public Health* 2012, **12:**613. 10.1186/1471-2458-12-613 PMC3444331 22867099

[50] Manzelmann RS, Passos JP (2010). Nursing images and representations concerning stress and influence on work activity. Rev Esc Enfem.

[51] Hackett RD, Bycio P (1996). An evaluation of employee absenteeism as a coping mechanism among hospital nurses. J Occup Organ Psychol.

[52] Henning R, Warren N, Robertson M, Faghri P, Cherniack M, Team C-NR: **Workplace health protection and promotion through participatory ergonomics: an integrated approach.** *Public Health Rep* 2009, **124:**26. 10.1177/00333549091244S104 PMC2708654 19618804

[53] Chan CW, Perry L (2010). Lifestyle health promotion interventions for the nursing workforce: a systematic review. J Clin Nurs.

[54] Blake H, Lee S (2007). Health of community nurses: a case for workplace wellness schemes. Br J Community Nurs.

[55] Milani RV, Lavie CJ (2009). Impact of worksite wellness intervention on cardiac risk factors and one-year health care costs. Am J Cardiol.

[56] Baicker K, Cutler D, Song Z: **Workplace wellness programs can generate savings.** *Health affairs* 2010**:**10.1377/hlthaff. 2009.0626. 10.1377/hlthaff.2009.0626 20075081

[57] Hassard J, Wang D, Cox T, Muyalert T, Flaspöler E: **Motivation for employers to carry out workplace health promotion.** [https://osha.europa.eu/en/publications/literature_reviews/motivation-for-employers-to-carry-out-workplace-health-promotion]

[58] Palumbo MV, Wu G, Shaner-McRae H, Rambur B, McIntosh B (2012). Tai Chi for older nurses: a workplace wellness pilot study. Appl Nurs Res.

